# Design, Use, and Effects of Sex Dolls and Sex Robots: Scoping Review

**DOI:** 10.2196/18551

**Published:** 2020-07-30

**Authors:** Nicola Döring, M Rohangis Mohseni, Roberto Walter

**Affiliations:** 1 Institute of Media and Communication Science Ilmenau University of Technology Ilmenau Germany

**Keywords:** sex toys, sexual objectification, anthropomorphization, embodied sexual fantasies, parasocial interactions and relationships, mobile phone

## Abstract

**Background:**

Although sex toys representing human body parts are widely accepted and normalized, human-like full-body sex dolls and sex robots have elicited highly controversial debates.

**Objective:**

This systematic scoping review of the academic literature on sex dolls and sex robots, the first of its kind, aimed to examine the extent and type of existing academic knowledge and to identify research gaps against this backdrop.

**Methods:**

A comprehensive multidisciplinary, multidatabase search strategy was used. All steps of literature search and selection, data charting, and synthesis followed the leading methodological guideline, the Preferred Reporting Items for Systematic Reviews and Meta-Analyses extension for Scoping Reviews (PRISMA-ScR) checklist. A total of 29 (17 peer reviewed) and 98 publications (32 peer reviewed) for sex dolls and sex robots, respectively, from 1993 to 2019 were included.

**Results:**

According to the topics and methodologies, the sex doll and sex robot publications were divided into 5 and 6 groups, respectively. The majority of publications were theoretical papers. Thus far, no observational or experimental research exists that uses actual sex dolls or sex robots as stimulus material.

**Conclusions:**

There is a need to improve the theoretical elaboration and the scope and depth of empirical research examining the sexual uses of human-like full-body material artifacts, particularly concerning not only risks but also opportunities for sexual and social well-being.

## Introduction

### Background

In 2050, it will be perfectly normal for women and men to experience love and sex with robots. This bold prediction from roboticist David Levy [1] started a debate, now more than a decade after, on the ethics, design, use, and effects of human-like, anatomically correct sex robots and of sex dolls, their noninteractive, immobile precursors. Futurologist Ian Pearson [2] went further by predicting that by 2050, women and men will have more sex with robots than with their conspecifics. One may question the validity of these predictions, but there is no doubt that technological change affecting all areas of life will not leave human sexualities unaffected. Significant changes in sexual behavior because of digital media and technologies are already well established [3,4]. Embodied technologies such as sex dolls and sex robots should not be overlooked in this context, especially as the popularization of the sexual uses of human-like material artifacts has long since begun.

So-called *sex toys* representing human body parts (eg, penis-shaped dildos and vibrators) are widely used and normalized. The lifetime prevalence of vibrator use, for example, is approximately 50% for heterosexual-identified women and men in the United States and Germany [5,6]. Sex toys are also popular in noncisgender and nonheterosexual populations [7]. Through web-based retailers, the sex toy market has expanded and diversified in recent decades, successfully targeting female customers in particular [8]. In the digital age, sex toys are becoming increasingly technologically advanced. Vibrators having integrated cameras are now available that can be remotely controlled by a partner in a long-distance relationship or that can synchronize with the user’s digital music playlist or preferred virtual reality porn. The development of innovative sex toys is, at least in part, pushed by customer demand. This is demonstrated by crowdfunding projects in which future customers grant investment money to entrepreneurs who bring new sex toys to the market (eg, the *Ambrosia Vibe*, a so-called *bionic dildo* providing haptic biofeedback while strapped on). There is also growing interest in the development of sex toys for aging populations and for people with disabilities, for instance, sex toys that are mind-controlled and therefore do not require hand function [9,10].

Despite the broad acceptance of sex toys representing human body parts, the development and marketing of human-like full-body sex dolls and of interactive and moving full-body sex robots have elicited great controversy in both public and academic discourses [11,12]. The discrepancies begin with the clarification of the central concepts. Should sex dolls and sex robots simply be regarded as next-level, high-end sex toys? Do they play a different and more important role in the sexual and social lives of their owners and users? Are they treated as surrogates for real partners or even accepted as fully adequate posthuman synthetic partners? This would raise questions not only about their impact on sexual and overall health but also about the future of intimate relationships.

### Domestic Use of Sex Dolls and Sex Robots

A typical usage scenario for sex dolls and sex robots is the domestic context in which the artifacts—after purchase—are available for recreational and long-term use at home. Some authors predict strong positive effects of sex dolls and sex robots, including social companionship, sexual exploration, pleasure, and increased satisfaction for individuals and couples [1]. Others, focusing on male users, predict strong negative effects in terms of objectification of and violence against women [13]. They assume that the men using women-like sex dolls or sex robots will be trained to sexually objectify real women and to disregard sexual consent. Furthermore, they predict that women and adolescent girls, already harmed by ubiquitous exposure to unrealistic beauty standards in the media, will feel even more inadequate when exposed to a consumer culture marketing perfectly beautiful, eternally youthful, and completely submissive female-gendered sex dolls and sex robots. Are we looking into an even more gender-unequal future?

Or are we just creating it with one-sided, male-centered, and sex-negative predictions? Why do we not ask different questions [11], such as: What do women want from innovative sex technologies? How could we design and market women- and couple-friendly, feminist, queer, empowering, and inclusive sexual health– and well-being–promoting sex dolls and robots? Most of the claims about current and future effects of sex dolls and sex robots are purely speculative so far because design studies and empirical use and effect studies are scarce.

### Commercial Use of Sex Dolls and Sex Robots

The same holds true for the commercial use of sex dolls and sex robots. The first so-called *sex doll brothels* have already opened in Asia, North America, and Europe, accompanied by strong media publicity (HJ Nast, unpublished data, 2019) [14]. In *sex doll brothels*, customers pay an hourly fee to be in a room with a human-like sex doll of their choice. Some authors argue that dolls and robots used as *surrogate prostitutes* are a good thing as they relieve women from prostitution and could reduce sexual violence [15]. However, we have not yet seen data collected from sex workers’ perspectives on the issue. Do they want to be relieved of their jobs or are they more afraid of dolls and robots as new competition? Furthermore, anecdotal evidence shows that some customers are now booking both a sex worker and a sex doll. This points to possible commercial use scenarios marked by neither substitution nor competition but collaboration between human sex workers and sex dolls or robots.

Again, the conceptualization is unclear here. What are the practical, legal, and ethical implications of framing short-term commercial use options as a *brothel* or *escort* instead of a *rental* business? Who are the customers—that is, will regular customers switch to dolls, or will we see new *technophilic* customers specifically requesting dolls or robots? Will demand for short-term commercial use of sex dolls and sex robots persist, increase, or wane? Budget restrictions (life-like sex dolls and robots are very expensive), need for discretion (hiding a full-body sex doll or robot from other household members is nearly impossible), and media-induced curiosity (seeing sex dolls and robots represented in pornography and fictional and nonfictional media can be intriguing) might be factors motivating a trip to the *sex doll brothel* today. Will these factors still play a role tomorrow when markets, media representations, and attitudes change?

Commercial short-term use of sex dolls and sex robots is barely understood, but is so highly controversial that some of the first *sex doll brothels* in Europe, North America, and Asia had to close shortly after opening because of community protests and police raids, as reported in numerous news media.

### Therapeutic Use of Sex Dolls and Sex Robots

The perspectives of clinicians are also divided. Some therapists, based on first case studies, explain how living with a *love doll* (as doll owners often prefer to call them) can be a helpful and healing transitional process after traumatic experiences, especially when accompanied by professional therapeutic care [16]. Other clinical authors warn their colleagues that products from the *sex robot industry* are marketed with health claims that are *rather specious* [17]. Even more heated are debates about childlike sex dolls produced in Asia and shipped worldwide. Some ethicists and clinicians argue that people with pedophilic preferences could use such dolls or robots as substitutes to prevent them from committing actual child sexual abuse and that therapeutic use might be promising [18]. Other ethicists and therapists completely reject this idea and warn that childlike sex dolls or robots are very harmful as they normalize and foster child sexual abuse in both pedophilic individuals and the society at large [19]. Legal bans against child sex dolls and robots are not only campaigned for (*Campaign against sex robots*) but, in some countries, also already in preparation or in effect (eg, the *Curbing Realistic Exploitative Electronic Pedophilic Robots Act of 2017—CREEPER Act of 2017* for short—in the United States) [20].

Again, conflicting approaches are visible in clinical, ethical, and legal debates. Should sex dolls and sex robots of all kinds be explored as possible therapeutic tools in the context of different paraphilic disorders and other sexual pathologies? Or should at least some of them be criminalized immediately, with the implication that new forms of doll- and robot-related sexual deviance have been introduced and must be prosecuted?

### Objectives, Questions, and Purpose of the Scoping Review

Against this backdrop of highly polarized debates, this scoping review study aimed to examine the extent and type of existing academic knowledge on sex dolls and sex robots and to identify gaps in theory and evidence as well as areas for further inquiry. In accordance with the leading methodological guideline for scoping reviews, the Preferred Reporting Items for Systematic Reviews and Meta-Analyses extension for Scoping Reviews (PRISMA-ScR) checklist [21,22], we will proceed to explain, separately and in detail, the review objectives, questions, and purpose.

#### Review Objectives

To comprehensively map the state of research on sex dolls and robots, it is necessary to cover academic literature from different disciplines and address various dimensions of the issue. Often, debates firstly and primarily focus on the negative or positive *effects* of sex dolls and sex robots. Effects are an important dimension of this issue. However, it is crucial to be aware that effects always depend on the users and *use* in different settings (eg, domestic, commercial, and therapeutic) as well as on the selected sex doll’s or sex robot’s *design* (eg, gender, age, race, body type, and sexual and nonsexual functionalities). Hence, this review, as indicated by its title, addresses the design, use, and effects of both sex dolls and sex robots.

#### Definition of Sex Dolls

*Sex dolls* are defined as human-like, full-body, anatomically correct anthropomorphic dolls of different materials (eg, rubber, plush, silicone, and thermoplastic elastomer) and price ranges that are designed for sexual use [11]. Sex dolls have at least one penetrable orifice (mouth, vagina, or anus) and/or one body part that can be inserted by the user (tongue or penis). The doll parts for sexual penetration or insertion are usually designed to be removable for cleaning. The special thing about sex dolls is their sexual functions, but this does not mean that they are used exclusively for sexual purposes. They can also serve as artificial love partners, social companions, or photo models, which is why their owners often call them *love dolls* or simply *dolls*. The term is also used by most scientists and parts of the media.

Sex dolls come in different genders (female, male, or trans), races (eg, white, Asian, or African), ages (adult, adolescent, or child), body types, skin, hair, and eye colors. High-end sex doll manufacturers (eg, RealDoll Abyss Creations, Sinthetics, and Orient Industry) offer ample options for selection and customization and also produce custom-made sex dolls. Therefore, *abstract sex dolls* with no resemblance to a specific real person need to be differentiated from *portrait sex dolls* designed in the likeness of a real person (eg, porn star, celebrity, or ex-partner). True-to-life sex dolls like RealDolls from Abyss Creations are delicate, need care and repair, and are not easy to handle because of their weight of approximately 65-70 lbs for female dolls and 85-105 lbs for male dolls.

Thus far, the sex doll market—determined by customer demand—offers mainly female sex dolls with highly sexualized looks that meet traditional feminine beauty standards (young, slim, pretty face, long hair, and large breasts). However, customization already allows for more body diversity (eg, androgynous or gender-queer looks), including the deliberate design of so-called bodily flaws (eg, moles, scars, stretch marks, belly fat, or body hair). Hence, the sex doll industry caters to different appearance-related customer demands (eg, the illusion of perfect supernatural beauty, resemblance to a real person, specific body-related preferences, or fetishes).

#### Definition of Sex Robots

*Sex robots (sexbots)* are defined as human-like, full-body, anatomically correct humanoid service robots of different materials, technologies, and price ranges that are designed for sexual use [11]. Sex robots look like sex dolls but are equipped with sensors, actuators, and artificial intelligence (AI). Sometimes, they are called AI sex dolls or robotic sex dolls to characterize them as upgrades of their noninteractive, immobile precursors. For gender sex robots, the binary terms fembot and malebot or gynoid and android are used. Sex robots come with all the attributes and functionalities of sex dolls and, in addition, can display conversation skills, emotions, and preprogrammed personalities. Furthermore, they can perform partially autonomous behaviors such as sexual movement (eg, hand movement for masturbation) or simulation of orgasm. However, the range of behaviors of existing sex robots is very limited. It can be assumed that the handling and maintenance of sex robots as large, heavy, and technically advanced products is demanding. Like sex dolls, sex robots are defined by their sexual functions but are also suitable for other functions in addition to sex (eg, social companionship).

Sex robots marketed today should not be confused with *concepts of future advanced sex robots* that are envisioned as having sentience, consciousness, free will, morality, and possibly even the legal status of citizens. There are also visions of future multifunctional assistance robots for domestic use that will do housework and errands, look after children, provide elderly care services, and offer sexual services. These imagined advanced sex robots or multifunctional robots with sexual functions appear in science fiction (eg, the Swedish television series *Real Humans* or the US movie *Ex Machina*) and in recent philosophical and legal sex robot debates [23,24], but are far away from the current state of technological development.

#### The Relevance of Sex Dolls and Sex Robots

Although high-end, true-to-life sex dolls have been on the market for more than 20 years (the leading US manufacturer Abyss Creations, creator of RealDoll, was founded in 1997), sex robots are still in a very early stage of development. The manufacturer TrueCompanion claims to have brought the world’s very first sex robots to the market. It presented its female-gendered sex robot *Roxxxy* to the public in 2010 and later announced the male-gendered sex robot *Rocky*, stirring a media frenzy [25]. However, it is assumed today, for good reasons, that *Roxxxy* and *Rocky* have never been more than overhyped prototypes [14,26]. Thus far, not a single customer has surfaced, and the TrueCompanion webshop has not changed over the years. The established RealDoll manufacturer Abyss Creations launched its first sex robot *Harmony* in 2018, followed by *Solana* and *Henry*. Sex robot *Samantha* by the Spanish manufacturer Synthea Amatus and sex robot *Emma* by the British-Chinese manufacturer AI Tech UK have likewise been sold since 2018. All these sex robots are sex dolls enhanced with some very limited AI and interactive features. Hence, although supposedly thousands of experienced sex doll owners exist worldwide, who have built their own distinct doll owner communities with online forums and offline meet-ups, there is, by comparison, only a very small number of pioneer users of sex robots. This limits the options for empirical research on long-term sex robot users, use, and effects.

However, as we are transitioning into the age of the robot, and sex robots provide interactivity, AI, and partly autonomous behavior, sex robots have been attracting much more public and scholarly attention than sex dolls. After all, they have been an integral part of science fiction literature for decades [27]. Considering the history of and relation between sex dolls and sex robots, it seems reasonable to address them collectively in this research review concerned with the sexual uses of human-like full-body material artifacts.

#### Review Questions

In mapping the current state of academic knowledge on sex dolls and sex robots, the scoping review aimed to answer the following 4 review questions (RQ):

RQ1: What is the state of sex doll and sex robot research in terms of the overall amount and type of research?

RQ2: What is (not) known about the design of sex dolls and sex robots?

RQ3: What is (not) known about the users and uses of sex dolls and sex robots?

RQ4: What is (not) known about the effects of sex doll and sex robot use?

These 4 RQs will be addressed separately for sex dolls and sex robots based on the respective literature searches.

#### Review Purpose

By systematically mapping the current state of academic knowledge on sex dolls and sex robots, this scoping review aimed to advance the understanding of sex researchers and practitioners and foster their professional involvement in the field of sexual uses of human-like material artifacts. Technicization and digitalization are fundamental societal processes that affect all areas of life, including human sexualities. Sex researchers and practitioners must be prepared to deal with these transformations in an informed and professional way, reflecting their own knowledge gaps, prejudices, and projections. Sex dolls and sex robots seem to be a particularly fruitful field of inquiry and professional development, as they often elicit very strong emotions that need to be recognized, worked through, and questioned with the help of clear conceptualizations, sound theories, and solid empirical evidence.

## Methods

A scoping review is “a form of knowledge synthesis that addresses an exploratory research question aimed at mapping key concepts, types of evidence, and gaps in research related to a defined area or field by systematically searching, selecting and synthesizing existing knowledge” [28]. As the body of academic literature on sex dolls and sex robots has not yet been comprehensively reviewed and exhibits a broad and heterogeneous nature that is not amenable to a more precise systematic review, a scoping review is of particular use [29]. Our procedure follows current methodological guidelines for conducting systematic scoping reviews [29], particularly the PRISMA-ScR checklist [21,22].

### Literature Search

To search for relevant academic publications on sex dolls and sex robots, the following 5 scientific literature databases covering different disciplines were used to ensure a multidisciplinary, multidatabase search strategy:

1. *Scopus* (largest academic literature database, approximately 57 million references, covering different disciplines, 1960-current),
2. *Medical Literature Analysis and Retrieval System Online (MEDLINE*; approximately 28 million references, focus on medicine, 1950-current),
3. *PsycINFO* (approximately 4 million references, focus on psychology, 1806-current),
4. *Institute of Electrical and Electronics Engineers (IEEE) Xplore* (approximately 4.5 million references, focus on technology, 1872-current), and
5. *Association for Computing Machinery (ACM) Digital Library*—Guide to Computing Literature (approximately 3 million references, focus on computing, 1950-current).

For sex dolls, the search terms “sex doll,” “sex dolls,” “doll sex,” “love doll,” “love dolls,” and “doll love” were used. For sex robots, the search terms “sex robot,” “sex robots,” “sexbot,” “sex bot,” “robot sex,” “love robot,” “love robots,” “lovebot,” “love bot,” and “robot love” were used. Search terms were applied to publication titles, abstracts, and keywords. Searches were limited to the English language, without publication date, publication type, or study type restrictions.

The search strategy was validated through the retrieval of a key set of relevant publications in Scopus, where 24 citations for sex dolls and 73 citations for sex robots were identified. The Scopus search strategy was then translated to the other 4 databases and executed between August 6, 2019, and August 9, 2019 (Multimedia Appendix 1 shows the full documentation of the electronic search strategy). Bibliographic information for all search results was exported from the databases into the citation management software Citavi 5.7.1 (Swiss Academic Software GmbH).

### Literature Selection

The literature selection included 3 steps: (1) the removal of duplicates among identified records; (2) the scanning of citations, titles, and abstracts for eligibility; and (3) the retrieval of full texts and assessment of eligibility. As we are reviewing an innovative emerging research field, we included all study and publication types from all available publication years. The only 2 exclusion criteria applied were lack of topical relevance (irrelevant were all publications that did not provide substantial knowledge about sex dolls or sex robots, that is, publications that only mentioned but did not investigate the topic or only referred to relevant publications) and nonaccessibility of published full text. We used the reference lists of all eligible full texts found through the databases to systematically search for further publications. Screening, assessing, and inclusion were performed in duplicate.

**Figure 1:**
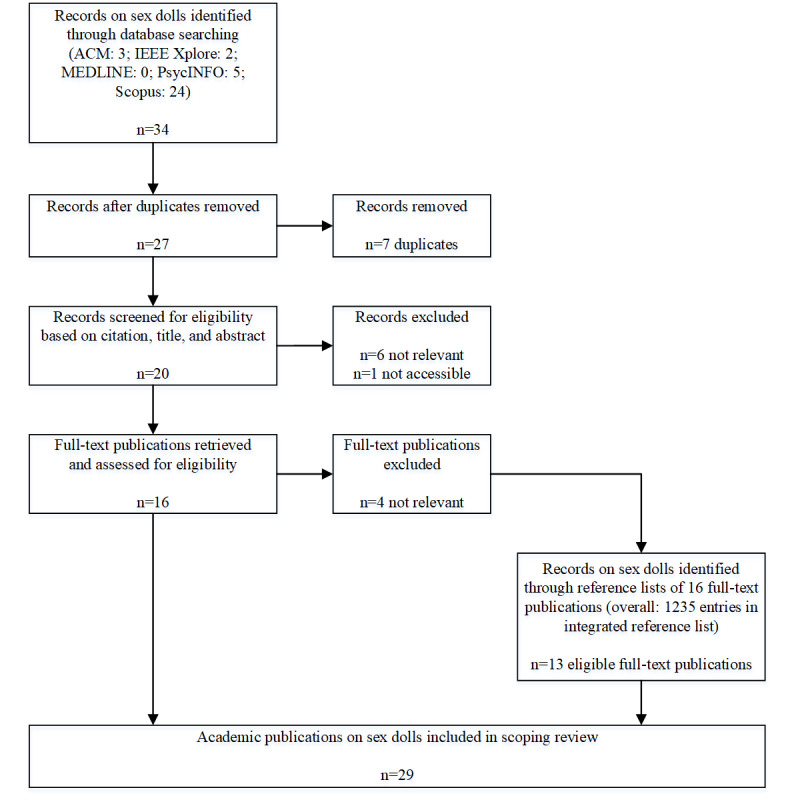
Flowchart of the scoping review procedure for literature identification and selection of sex dolls. ACM: Association for Computing Machinery. IEEE: Institute of Electrical and Electronics Engineers; MEDLINE: Medical Literature Analysis and Retrieval System Online.

As can be seen in Figure 1, for sex dolls, we identified 16 eligible publications through the databases and 13 additional publications through their reference lists, resulting in 29 included sex doll publications (Figure 1).

**Figure 2:**
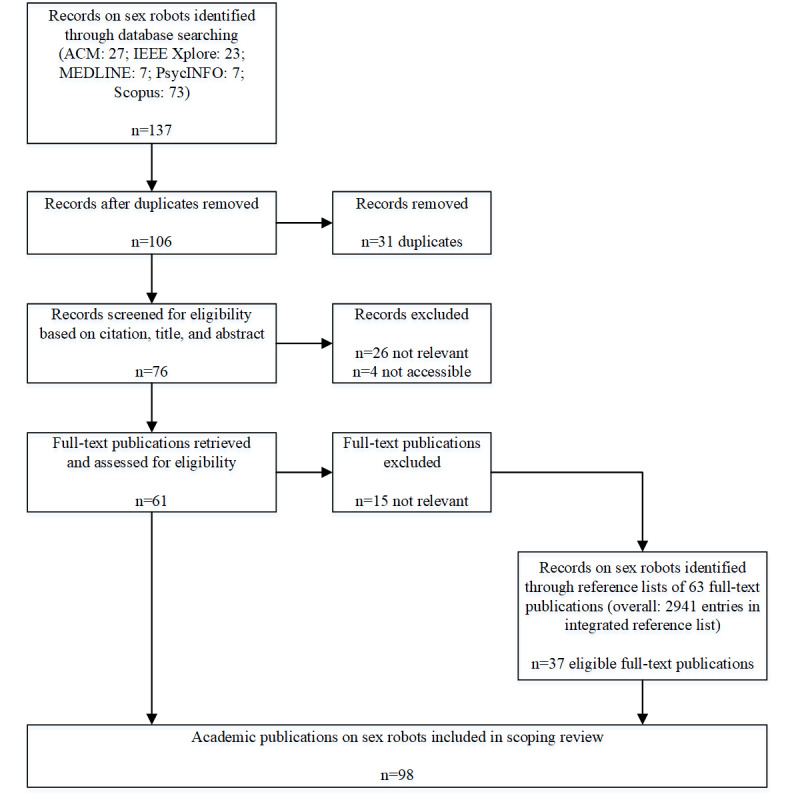
Flowchart of the scoping review procedure for literature identification and selection of sex robots. ACM: Association for Computing Machinery; IEEE: Institute of Electrical and Electronics Engineers; MEDLINE: Medical Literature Analysis and Retrieval System Online.

The same procedure was used for sex robot publications, resulting in 98 included publications (Figure 2).

### Data Charting

During the data charting phase, all included publications were reviewed and charted using a data charting form that was pilot tested for all 29 included sex doll publications, discussed within the team, and revised 3 times. The final charting form has a sex doll and a sex robot version, each including 10 variables: (1) citation (author and year), (2) citation count (derived from Google Scholar), (3) publication type, (4) peer review, (5) academic discipline (derived from the first author’s academic position and/or education), (6) study type (derived from topic and methodology and used for grouping of sex doll/sex robot publications), (7) key findings regarding the study’s research question, (8) key findings—if applicable—regarding sex doll/robot design, (9) key findings—if applicable—regarding sex doll/robot use, and (10) key findings—if applicable—regarding sex doll/robot effects. Variables 1 to 7 were used to answer RQ1 in the overall state of the research, variable 8 addressed RQ2, variable 9 addressed RQ3, and variable 10 addressed RQ4. The data were charted in duplicate.

### Synthesis and Reporting of Results

First, a numeric overview of the number, type, and distribution of included publications was created using 2 summary tables and a chart of the timeline of publication activity. Second, a narrative synthesis of the results of the previous studies was created, focusing particularly on their insights regarding design, use, and effects of sex dolls and sex robots. Third, to fully answer the RQs on “what is (not) known” about sex dolls and sex robots, the state of research was critically assessed for research gaps, and recommendations for future research were included. To avoid vagueness and to achieve maximum usefulness, recommendations for future research with specific references to applicable theories, relevant methods, and related research fields were backed up. All steps of data synthesis and reporting were discussed within the team and performed in duplicate.

## Results

### State of Research on Sex Dolls

To summarize the state of research on sex dolls, we first map the number and type of publications and then report their main results regarding the design, use, and effects of sex dolls before coming to the research gaps and recommendations on how to fill them.

#### Amount and Type of Research on Sex Dolls

During the scoping review literature identification process, we included 29 academic publications on sex dolls (Figure 1). This body of literature consists of 5 distinct groups of studies according to both their topics and methodologies, which are closely linked (Table 1).

**Figure 3:**
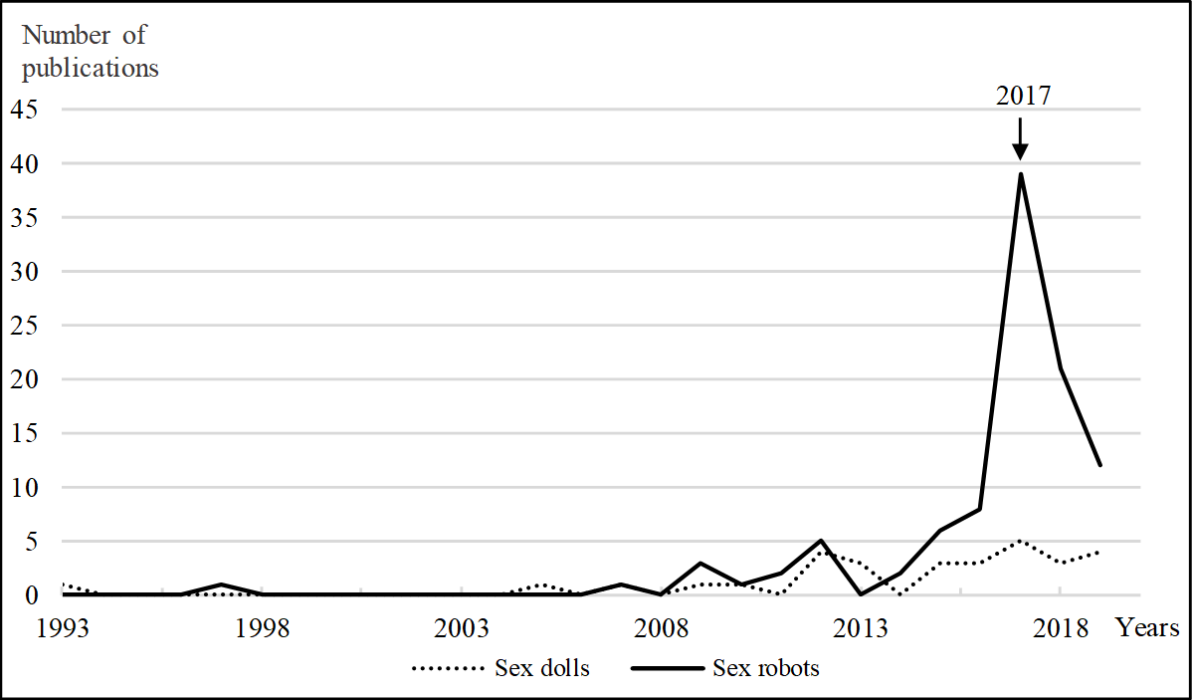
Timeline of publications for sex dolls (N=29) and sex robots (N=98).

The body of academic literature contains 2 published monographs that exclusively focus on sex dolls [33,43]. Approximately 50% (17/29) of the included sex doll publications were peer reviewed. The Google Scholar citation count reveals a range from 0 to 46 citations; the most cited publication was the monograph *The Sex Doll: A History* by Anthony Ferguson [33]. It is noteworthy that all of the most cited publications within each of the 5 groups of sex doll publications were not peer reviewed. Regarding the timeline, the oldest sex doll publication identified in the databases and included in our review is a 1993 clinical case study from medicine [50] that deals with the shared use of an inflatable sex doll. However, this is an outlier, with >85% (25/29) of the sex doll publications having been published within the past 10 years (2010-2019; Figure 3).

**Table 1:** Amount and type of research on sex dolls (N=29 included academic publications, based on literature search in August 2019).

Reference		Citation count^a^	Peer review	Academic discipline
**Sex doll conceptualization and theory (n=11)**				
	Blizzard (2015) [30]	—^b^		Science and technology studies
	Blizzard (2018) [31]	0		Science and technology studies
	Cassidy (2016) [32]	5	✓	English
	Döring and Pöschl (2018) [11]	6	✓	Psychology
	Ferguson (2010) [33]	46		Unknown
	Kim (2012) [34]	5	✓	Women’s and gender studies
	Levy (2012) [14]	27		Artificial intelligence
	Nast (2017) [35]	7	✓	International studies
	Nast (2019) (HJ Nast, unpublished data, 2019)	—	✓	International studies
	Ray (2016) [36]	3		English
	Wong (2015) [37]	0		Sociology
**Sex doll representations in art and media (n=7)**				
	Burr-Miller and Aoki (2013) [38]	7	✓	Communication and media studies
	Connor (2015) [39]	2	✓	English
	Getsy (2013) [40]	6	✓	Art history
	Koné (2016) [41]	0		German
	Roos (2005) [42]	9	✓	English
	Smith (2013) [43]	21		Visual arts
	Weisel-Barth (2009) [44]	1	✓	Psychoanalysis
**Empirical studies on sex doll use and effects (n=5)**				
	Ciambrone et al (2017) [45]	5	✓	Sociology
	Knox et al. (2017) [46]	3	✓	Sociology
	Langcaster-James and Bentley (2018) [47]	1	✓	Anthropology
	Su et al (2019) [48]	0	✓	Human-computer interaction
	Valverde (2012) [49]	15		Psychology
**Clinical case studies on sex doll use and effects (n=3)**				
	Kleist and Moi (1993) [50]	18		Medicine
	Knafo (2015) [16]	3	✓	Clinical psychology/psychoanalysis
	Knafo and Lo Bosco (2017) [51]	12		Clinical psychology/ psychoanalysis
**Legal regulation of child sex dolls (n=3)**				
	Brown and Shelling (2019) [19]	0	✓	Criminology
	Chatterjee (2019) [52]	0	✓	Criminology and law
	Maras and Shapiro (2017) [20]	7		Criminology and law

^a^Citation count according to Google Scholar in August 2019.

^b^Google Scholar did not list the reference.

#### Research Findings on Sex Dolls

We have summarized the main findings of previous sex doll research separately for the 5 groups of sex doll publications (Table 1).

#### Sex Doll Conceptualization and Theory

The largest group of sex doll publications (11/29, 38%) consists of theoretical studies that aim to conceptualize the human–sex doll relationship. Half of them (6/11) promoted a critical feminist conceptualization of the female sex doll as an expression and affirmation of patriarchal gender power relations and women’s sexual objectification by men (ie, by male sex doll producers, owners, users, and observers). The publications deal with the production and use of female sex dolls in Western [30,32,33,36] and Asian (HJ Nast, unpublished data, 2019) [35] countries and sometimes discuss gender issues of female sex doll use in relation to economic, cultural, and racial issues as well as in relation to recent crises of masculinity. Their overall assessment of female sex dolls and their effects is very negative. A typical example of the critical feminist conceptualization of female sex dolls is as follows [33]:

The female sex doll is man’s ultimate sexually idealized woman. It is never more than the sum of its fully functional parts. A woman rendered harmless, it is immobile, compliant, and perhaps most importantly, silent. What the user of the sex doll seeks is the negation of change and the comfort of always retaining control of the relationship.

The other half of the theoretical papers (5/11) conceptualize human–sex doll relations, mainly in a positive way [11,14,31,34,37]. These papers do not limit their focus to female dolls or (supposedly heterosexual, sexist, and misogynist) male doll users only, but they address the already observable and potentially growing diversity of both dolls and doll users (eg, including women, queers, older people, and people with disabilities). Furthermore, they reject the 2 key assumptions of the critical feminist conceptualizations that dolls are inanimate objects for mere (and questionable forms of) male sexual gratification (eg, acting out sexual fantasies of subjugation and violence against women) and that they are surrogates for real women. Instead, dolls are conceptualized as *new types of social actors*, neither inanimate objects nor surrogate humans but as *posthuman partners* or as *interanimated beings* [31,34,37].

What dolls *are* and what human-doll relationships *mean* is, therefore, not predefined by attributes of the doll, but is the result of the *connections* between the human beings and the *doll beings*. It is up to the users if they *abuse* or *take care of* their dolls if they act out hatred or love. The anthropomorphic, anatomically correct full-body doll in this context might appear passive. The papers, however, argue that in its passivity lies agency and even power [34]: The *doll being*, although vulnerable to abuse just as the human being, is easily able to elicit attention, care, love, and long-term relationships. The conceptualization of dolls as interanimated beings covers rather than denies situations of doll objectification and abuse. However, it also covers situations of doll appreciation, care, and love. Most importantly, such a conceptualization covers complex situations of mixed and ambivalent connections between dolls and their users.

#### Sex Doll Representations in Art and Media

The second largest group of sex doll publications (7 out of 29; Table 1) analyzes sex doll representations in art and media. Several studies explain that men creating idealized and sexualized female statues, mannequins, or dolls is a *common trope* in the history of art and culture that can be understood as an expression of patriarchal gender relations, objectification, and fetishization of women [39,42,43]. One notorious example is Ovid’s poem about the sculpturer and ancient Greek mythical king of Cyprus, *Pygmalion*. Mythical Pygmalion, appalled by female sexual permissiveness, turned away from real women and created an ivory sculpture of his ideal woman. He physically loved the sculpture, and it later came to life.

A very famous example from modern cultural history is the Austrian artist *Oskar Kokoschka*, who in 1919 commissioned an anatomically correct sex doll in the likeness of his former lover Alma Mahler after she had ended both the relationship with him and her pregnancy. The *Alma Mahler doll* is an example of a portrait sex doll produced without the consent of the person portrayed. Kokoschka lived with the Alma Mahler doll, hired a maid for her, brought her to public spaces like the opera, and created numerous drawings and paintings of her before he destroyed the doll [42,53]. Kokoschka’s strange and scandalous actions were often dismissed as a private matter of grief, trauma, or insanity. However, they can also be read as an expression of male entitlement and an attempt to exercise revenge by publicly shaming Alma Mahler. Last but not least, according to the literature, there is also good reason to consider this case as some sort of performance art [42]. Within the sex doll literature, the Alma Mahler portrait sex doll is addressed the most in papers interested in sex doll representations in art and media (4 out of 7) [39,41-43] but is also mentioned in theoretical [33,37], empirical [49], and case study [16,51] sex doll publications.

Although the feminist critique of sex dolls plays a role, most publications in this group provide more complex interpretations. They point to the fact that in creating sexualized female dolls, male artists deal with more than gender relations, also dealing, for example, with their own fear of death [39] or with their own object status [42]. Furthermore, some authors in their cultural analyses point to sex doll–related artwork that reveals additional and potentially emancipatory dimensions of sex doll use. An artist in the United States, Amber Hawk Swanson, who identifies as a lesbian commissioned a RealDoll sex doll in her own likeness from Abyss Creations, married her, and lived and collaborated with her in video and performance artwork. Amber Swanson’s *Amber Doll Project* (2006-2011) triggered and disrupted the audience’s clichéd (heterosexual) fantasies about lesbian desire, twin sexuality, and the role of females as sexual objects [40].

Another cultural analysis stresses the 2 main functions of dolls: they are made to be looked at and to be played with [41]. Although a feminist critique often assumes a rigid misogynist meaning and use of female sex dolls, art projects demonstrate more complex, creative games to be played with dolls. An artist in the United States Laurie Simmons brought back a female sex doll from Japan and created a series of photographs of her. *The Love Doll* (2009-2011) project goes beyond affirmation and deconstruction of sexual objectification as the female artist casts a loving, *maternal gaze* on her doll, thus inventing “a novel game to play with the doll” [41].

The last 2 publications deal with the representation of sex dolls in movies and television. The first one, the US movie *Lars and the Real Girl* (2007), is interpreted from a psychoanalytic perspective as an “inspiring tale of healing” [44]. The movie tells the story of withdrawn single 28-year old *Lars* who starts living with sex doll *Bianca* to end his loneliness. His family and the whole town play along by treating the doll as his legitimate partner and welcoming her as a new community member. This magically transforms everyone for the better. Ultimately, Lars can let go of the doll and turns toward a real woman. The movie deals with the contested topic of men’s relations with sex dolls in a very empathetic and romantic way. Interestingly, fictional Lars never has sex with his doll because *Bianca* is very religious, and thus, premarital sex is unthinkable. Tellingly, the acceptance of the *Bianca* doll by both the fictional community in which Lars lived and by the mainstream cinema audience required her to be a sexually abstinent sex doll [44].

The British Broadcasting Corporation (BBC) documentary *Guys and Dolls* (2007) portrays 4 men (*Davecat*, *Everard*, *Gordon*, and *Mike*) living with their female sex dolls. As media analysis reveals [38], the documentary explains the doll owners’ unusual lifestyle as a result of their *heteronormative shortcomings*. The heterosexual men were not able to create relationships with real women and hence settled with dolls. However, a queer reading of the documentary is also possible, as the lifestyle of a doll lover allows men to express their sexualities differently. In the context of doll care, a variety of feminine connotated sensual activities are legitimized and carried out (eg, washing, drying, powdering, dressing, and putting make-up on the doll). Thus, the documentary unintentionally illustrates that the doll owner identity can also be read as a *queer sexual identity* [38].

#### Empirical Studies on Sex Doll Use and Effects

The third group of sex doll publications (5 out of 29; Table 1) contains empirical studies on sex doll use based on potential future or on current doll users’ subjective accounts. We could not find empirical papers dealing with the prevalence of sex doll use. As reported in one of the above-cited theoretical papers [11], in a national web-based survey conducted in 2016 in Germany (N=2000; 50% female, 18-69 years), the lifetime prevalence of sex doll use was 9% for men and 2% for women. A web-based survey of 345 (81% female) undergraduate students in sexuality courses at a university in the United States revealed that 8% of the respondents would use a sex doll and 17% could understand a sex doll user [46]. The authors interpret the result as an indicator of the widespread stigmatization of sex doll use as opposed to the widespread acceptance of sex toy use.

To survey doll owners on their first-hand, long-term experiences with sex dolls, some researchers successfully turned to sex doll owner online community forums for recruitment. A psychological survey with 52 doll owners (6 female) of an English language international online doll owner forum showed that respondents used their dolls for solo and partnered sexual activities and evaluated the sexual experiences with their dolls as enjoyable [49]. Contrary to common belief, the surveyed doll owners (mean age, 43 years) did not show below-average mental health or life satisfaction on standardized scales; however, they reported possibly above-average problems with sexual functioning. Human-doll relationships are not always monogamous. A considerable number of surveyed male doll owners were in a relationship with a human partner (21%) and/or had more than one doll (39%). The author calls for more research on the psychologically adaptive and maladaptive uses of sex dolls.

An anthropological survey with 83 members (3 female, 2 gender fluid, 2 trans, and 1 other gender) of 2 English language international online sex doll forums revealed that most respondents characterize the relationship with their doll as a *sexual relationship* (50/83, 77%). At the same time, many respondents also describe their relationship with the doll as “companionship” (47/83, 57%) and as a “loving relationship” (39/83, 47%). The researchers conclude that so-called sex dolls not only provide sexual gratification but also serve as multifunctional dolls (they suggest the label *allodolls*) that can provide *posthuman kinship* and alleviation of loneliness.

Instead of using survey methodology, 2 other studies chose a nonreactive approach and collected sex doll owners’ publicly available web-based content. One study qualitatively analyzed 68 customer testimonials (4 written by females) published between 2006 and 2016 on the website of the RealDoll manufacturer Abyss Creations [45]. The researchers found that dolls foster the commodification of female bodies because (1) the manufacturer offers many options for customization that are in line with stereotypical beauty standards, and consequently, (2) the users write a lot and in great detail about their preferences regarding the bodily appearance of their female dolls. Apart from the physical beauty of the doll, emotional closeness to the doll also plays an important role in doll owners’ testimonials. They write extensively about the comforting effect of the doll’s mere presence [45]:

My doll arrived four days ago and my home has a new, warm feeling to it.

They also stress how much they enjoy taking care of the doll (doll maintenance includes regular washing, drying, powdering, and dressing) and, thus, feeling needed [45]:

She’s coming to life for me, I want to take care for [sic] her all the time. Yes, my life has become much fuller.”

The authors’ assessment of men’s attachments to sex dolls is ambivalent—concern about objectification of women’s bodies is mixed with acknowledgment of the creation of supportive emotional intimacy.

The same main result is reported by a qualitative content analysis of 316 discussion threads with 7775 posts from the Abyss Creations RealDoll online forum [48]. Sex doll owners create *embodied intimate fictions* with the dolls. They often praise their idealized bodily attributes and supernatural feminine beauty. However, they do not reduce the dolls to mere sex toys but create rich narratives (in both text and photographs) about their dolls’ personalities, backstories, and experiences, integrating domestic life, outdoor trips, and sexual encounters. Furthermore, the lively discussions in the online forum illustrate that doll owners not only bond with their dolls but also with other members of the doll owner community. As hobbyists, not unlike pet owners, they share tips and tricks around doll purchase, doll care, and doll photography.

#### Clinical Case Studies on Sex Doll Use and Effects

The fourth group of sex doll publications involves 3 clinical case studies, 2 from psychology, and 1 from medicine (3 out of 29; Table 1). The clinical-psychological case study of psychoanalyst Danielle Knafo [16] deals with a *48-year-old psychotherapy patient Jack*, an actuary by profession. He had suffered a problematic childhood with a derogatory mother, and his 2 marriages had failed. Deeply hurt by the most important women in his life and inspired by an online forum, he had bought RealDoll *Maya* for over US $10,000. Reluctantly, he shares with his psychoanalyst that *Maya* has now been his *girlfriend* for 2 years and that she is “beautiful” and “super in bed”. He adds how much he enjoys her company, how harmonious their relationship is (“we never fight”), and that he thinks he might be in love with her. However, he is also conflicted about his unusual lifestyle and therefore seeks therapeutic help. The feminist identified psychoanalyst reports how she was initially repulsed at the idea of a man choosing a sexist object as his girlfriend [16]. However, her “own perversity kicked in” along with a kind of “voyeuristic curiosity,” as she describes it [16]. She manages not to judge Jack but to understand him. She concludes that RealDoll *Maya* is more than a “perversion” and that she is an “invention” and a “lifesaver for Jack” [16]. During psychoanalysis with an accepting female therapist, Jack gains enough self-confidence and optimism to retire *Maya* and return to relationships with real women. In this case study, the sex/love doll served as a soothing and healing *transitional object* in the sense of Donald Winnicott’s [54] theory of transitional objects.

The second case study from the sex doll literature comes again from Danielle Knafo [51] and is based on 7 hours of personal interviews that she led with *Davecat, a 42-year-old African American self-proclaimed doll lover* in his Michigan home. Davecat has lived with RealDoll *Sidore Kuroneko* (nickname *Shi-chan*) since 1998 and regards her as his wife. They wear matching wedding rings inscribed with the words “Synthetic love lasts forever” [51]. In 2012, Davecat ordered a second doll, this time from the Russian manufacturer *Anatomical Dolls* and named her *Elena Vostrikova* (nickname *Lenka*). *Elena* has the status of a “mistress, plaything and companion” for both Davecat and his bisexual synthetic wife *Sidore*. *Elena* is built lighter with looser joints. “Elena is more built for sex whereas Sidore is built for love” as Davecat puts it [51]. *Muriel Noonan* (nickname *Mew-Mew*), his third doll, is made of wood, leather, Lycra skin, and cotton batting. She is least used for sex and mostly serves as a *flatmate*. Davecat has given all of his dolls complex backstories and personalities and lives with them in what he describes as a harmonious polyamorous *family* to which, at the time of the interview, he plans to add 2 more dolls in the future. Davecat explains how he experiences sex with a doll (for him a “synthetik [sic] partner”) in comparison to sex with a human (for him an “organik [sic] partner”) [51]:

Dolls overall are simultaneously robustly made and fragile. They’re ostensibly made for sex, but they’re also sculpture pieces. With an organik partner, obviously you can be a bit rougher, but I take care to be gentle with Shi-can and Lenka when we’re in bed. Another remarkable difference between organik and synthetik women is that when you’re inside a doll’s vagina or anus, there’s a vacuum effect that’s pretty… breathtaking. I’d say sex flows a little better with an organik, as she’s able to move herself, whereas changing positions with a doll requires you to pause and rearrange everything. Overall, though, personally, I’d rate sex with a synthetik woman to be as good, if not better, than with an organik woman. Mainly as a doll’s artificiality is a huge draw with me....

The psychoanalyst characterizes Davecat as a man who has been struggling all his life with intimacy issues and has found dolls as a viable alternative to having a human partner. At the same time, she acknowledges that Davecat feels sexually and emotionally attracted to the artificial aspects of dolls [51]. He self-identifies as an *iDollator*, a doll lover who prefers dolls to humans. This identity is so meaningful to Davecat that he serves as an activist and spokesperson for the doll lover community. He has participated in numerous press interviews, photoshoots, and television documentaries (eg, the earlier mentioned BBC documentary *Guys and Dolls*). The psychoanalyst, dissecting Davecat’s biography, neither stigmatizes nor pathologizes him. Although she assumes that his unconventional lifestyle is rooted in anxieties and conflicts [51], in her evaluation, it appears to be a viable solution. After all, Davecat is not harming anybody, is able to work, well-integrated socially, and satisfied with his life.

The third and last case study is a medical one. It proves that the shared use of an inflatable sex doll can lead to the transmission of a sexually transmitted disease (ie, gonorrhea) if the doll is not cleaned or no protection is used [50]. In this case, a male sailor had found the sex doll of a colleague on board by chance and used it secretly.

#### Legal Regulation of Child Sex Dolls

The fifth and final group of sex doll publications covers 3 publications on child sex dolls (3 out of 29; Table 1). All 3 call unanimously for a legal ban and explore the implementation of such a ban in different legal systems, namely, in Australia [19], the United Kingdom [52], and the United States [20]. They reject the idea of possible therapeutic value and stress that the production, marketing, and use of child sex dolls would normalize and foster child sexual abuse. The publications point to different harmful uses of child sex dolls (eg, the use of child sex dolls during grooming or during the abuse of children or the exploitation of individual children by producing portrait sex dolls in their likeness). The most important reason given for banning child sex dolls is the assumption that acting out child sexual abuse with a doll would rehearse, train, and trigger real child sexual abuse. Abstract child sex dolls are compared with computer-generated or so-called *fantasy child pornography* [52]. In both cases, no children are directly harmed in the process of production, but the dissemination and marketing of two-dimensional or three-dimensional depictions of sexualized children is still considered harmful and exploitative and should therefore be criminalized according to all three studies.

#### Research Gaps in Sex Dolls

There is a considerable discrepancy between the great media interest in the topic of sex dolls and sex robots mentioned in the introduction and the limited amount of scientific knowledge. Overall, the interdisciplinary field of sex doll research is fairly small (RQ1). *Empirical and clinical* studies on doll use, in particular, are scarce (5 peer-reviewed papers in total) and often have limited generalizability due to small convenience samples or single case studies. Accordingly, it is not surprising that many research gaps exist.

#### Research Gaps in Sex Doll Design

Regarding *sex doll design* (RQ2), many publications agree that the sexualized and idealized looks of female sex dolls pose a problem in terms of further sexual objectification of women within a patriarchal consumer culture already saturated with unrealistic beauty standards for women’s bodies. However, previous research falls short on conceptualizing the *sexual fantasy dimension of sex dolls*. Understanding dolls as embodied sexual fantasies, it is neither surprising nor questionable that dolls do not mirror reality as it is, or as it ethically ideally should be, but unapologetically express unrealistic, exaggerated, clichéd, and thus exciting and satisfying fantasies. Research on sexual fantasies has revealed that humans of all genders are usually not particularly turned on by morality or normality but often by the direct opposite [55,56]. What technological change brings about is ample new possibilities to express and materialize sexual fantasies formerly enjoyed purely privately so that they now become readable, audible, visible, and—with dolls and robots—even tangible in the public realm.

Although a sex-positive perspective usually acknowledges the value of fantasy, creativity, play, provocation, and pleasure, a critical perspective usually warns against the expression and dissemination of fantasies whose content is not in line with the ethical standards applied to real life. Obviously, child sex dolls are regarded as a hard limit in the academic sex doll literature. However, for other types of fantasies that dolls can and could embody, there is no consensus and not even a rational debate.

If the breast size of female sex dolls poses a problem (many authors complain about the female dolls’ *pornographic looks*), what range of breast sizes would be ethically correct and/or harmless enough regarding the prevailing beauty norms for female bodies? Do we need size norms for ethical dildos and vibrators as well? Questions like these are both banal and profound at the same time: meaningful critical evaluations of sex doll design should go beyond the trivial observation that sex dolls look like sexual clichés because that is exactly the point of sexual fantasy products. Young-looking sex dolls and related products like full-body cushions depicting sexualized young women (so-called *dakimura*) are often criticized, but, in Japan, for example, their main target group is young men and adolescent boys [35]. Is it inappropriate that they desire same-age dolls? Do we want older-looking dolls to be marketed to them? Racial issues are also very confusing. Regarding racial prejudices and privileges, one might problematize that in Japan, for example, exclusively Japanese-looking sex dolls that emphasize skin whiteness are marketed [35], whereas one may also problematize the marketing of Japanese-looking sex dolls to non-Japanese customers. Is there any way to criticize (and improve) sexual fantasy product designs and marketing strategies that take into consideration both the concern about social inequalities and vulnerabilities and the concern for sexual rights and freedom of sexual fantasy and expression?

The same issues have been discussed for decades regarding pornography [57]. Although some authors still claim that all pornography is inherently inhumane and sexist, just as some authors and activists claim that all sex dolls are inherently inhumane and sexist, other authors and activists accept that sexual explicitness and lack of realism are necessary ingredients of sexual fantasy products. However, they push for a greater variety of fantasies to be represented in the products. This is why female-friendly, couple-friendly, feminist, and queer pornographies have been produced and investigated since the 1980s [58]. The sex doll market could also be diversified. Exploring directions for diverse sex doll designs and their implications could be a task for future research. Design studies could bring together sex researchers, current and future customers with different gender and sexual identities and lifestyles (including older people and people with disabilities), sexual health experts, designers, and/or industry representatives. Collaborations with the sex doll industry promise new insights and, against common belief, do not imply the abandonment of critical analysis. Indeed, critical analysis is often much sharper and more to the point if researchers are closely familiar with the research subject and its context instead of only looking at it from a distance.

#### Research Gaps in Sex Doll Use

Although pornography use has become mainstream among men and women, it is unclear how large the sex doll user population is and whether it will grow or stay a niche market. Systematic analyses of market data and representative surveys of national populations regarding the prevalence and acceptance of sex doll use are widely lacking. In China, for example, due to the former one-child policy, there is a demographic surplus of millions of men—will they become a target group for sex dolls (HJ Nast, unpublished data, 2019)? With aging societies and a persistent gender gap in life expectancy, we will see a surplus of millions of widows and single older females in developed countries—perhaps another target group for sex dolls and further innovative sexual technologies.

Previous studies on sex doll owners’ experiences have demonstrated that men create complex, multi-dimensional relationships with their dolls that include, but are not limited to, the search for sexual gratification. To further explore the *psychology and sexuality of doll play and human-doll relationships*, theories, methods, and results from related research fields should be considered.

Although sex doll conceptualization struggles with the passivity and object status of dolls and the one-sidedness of human-doll relationships, in the field of media research, the concept of one-sided “parasocial relationships” between humans and media figures has been well developed for more than half a century [59]. It is also established that parasocial interactions and relationships are linked to well-being [60]. Romantic and erotic relationships between humans and media figures are common (eg, adolescent girls falling in love with members of boy groups from the music industry) and psychologically meaningful and helpful [61]. Established measures for parasocial interactions and relationships between humans and media personas could be adapted to investigate human–sex doll interactions and relationships.

Surprisingly, research on men’s play with female sex dolls has widely ignored the research on children’s play with childlike dolls and research on women’s play with babylike dolls. Children love, kiss, cuddle, talk to, and sleep with their dolls, and sometimes, they poke their dolls’ eyes, cut their hair without consent, or open their stomachs during questionable operations [62-64]. However, nobody assumes that children’s use of childlike dolls makes them antisocial or encourages them to treat other children like objects. The same holds true for the female adult doll owner community that uses realistic baby dolls (so-called *Reborn Dolls,*). Here, women use doll play to express sexuality-related fantasies of procreation and motherhood without being accused of antisocial inclinations or objectification of babies, although their behavior is criticized and scandalized in the media [51,65]. Last but not least, research on sex doll use could learn from research on so-called *doll therapy* [66]. Doll therapy addresses dementia patients and encourages holding, kissing, cuddling, talking to, feeding, or dressing an anthropomorphic doll because interactions and relationships with dolls provide comfort, control, and peace as well as feelings of pride, purpose, and bonding that can alleviate agitation and other symptoms [66]. Such soothing and healing effects of dolls have also been reported by sex doll owners. Theoretical elaboration is needed to link and/or differentiate the various user groups and uses of different types of dolls. Why is men’s play with sex dolls so outstanding in its assumed connections with antisocial tendencies and an unhealthy confusion of play and reality? Are male gender and sexual fantasy dolls such a dangerous coupling and/or are we dealing with sex-negative and gendered projections?

The previous literature points to different types of sex doll owners like the passionate, possibly paraphilic, lifelong *iDollator*; the misogynist doll owner, the possibly sadistic doll owner striving for complete dominance; the pedophilic doll owner; the transient doll user working through hurt and heartbreak or through teenage angst; the unattractive, old, or disabled user with very limited prospects of success in the real partner market; the doll photographer and hobbyist; or the sexually experimental female user and couple. However, a definitive typology is missing. According to the literature, approximately 20% of the sex doll owner community are couples and females [67], and thus far, we do not know much about them.

#### Research Gaps in Sex Doll Effects

Sex doll effects of both long-term and short-term sex doll use are under-researched. Long-term domestic use by doll owners has only been explored with small convenience samples and mostly without the use of established and validated measures for predictors and outcomes of sex doll use, for example, measures of sexual and mental health, personality, sociability, sexism, doll-related paraphilias (eg, objectophilia and doll fetishism), and new sexual identities (eg, digisexuality) [68]. Short-term commercial uses of sex dolls and their effects are completely unexplored. Interviews with customers of *sex doll brothels* and expert interviews with sex doll brothel staff could be helpful. The therapeutic uses and effects of sex dolls have also been under-researched. More clinical case studies are necessary.

What is special about sex dolls as sexual fantasy products is their materiality: they are embodied sexual fantasies, and their use demands specific sexual skills–fantasy skills to enrich the parasocial interaction and practical skills in positioning and moving the heavy doll to create an enjoyable and satisfying sexual experience. Thus far, no observational or experimental studies of social or sexual interactions between humans and sex dolls and their outcomes have been conducted.

#### State of Research on Sex Robots

We have summarized the state of research on sex robots by mapping the number and type of publications, reporting their main results and indicating the research gaps.

#### Amount and Type of Research on Sex Robots

During the scoping review literature identification process, we included 98 academic publications on sex robots (Figure 2). This body of literature consists of 6 distinct groups of publications according to both their topics and their methodologies (Table 2). The groups of sex robot publications are similar to those of sex doll publications, the main difference being unavailability of clinical case studies for sex robot, but the availability of many ethical studies and some design studies.

The largest group of sex robot publications (40/98, 41%) deals with sex robot conceptualization and theory, written by authors from social and life sciences, humanities, philosophy, and engineering. The second largest group of publications (28/98, 29%) addresses the ethics of sex robots and is mainly rooted in philosophy. The third group of publications contains empirical studies on sex robot use and effects (12/98, 12%), mainly from the fields of psychology and human-computer interaction. The fourth group of publications addresses sex robot representations in art and media (8/98, 8%), the fifth group of publications looks at child sex robots and their legal regulation (6/98, 6%), and the sixth and final group of publications involves sex robot design studies (4/98, 4%).

The body of academic literature contains 3 published monographs focusing exclusively on sex robots [1,69,70]. Approximately one-third of the included sex robot publications are peer reviewed (32 out of 98). Many sex robot publications are papers from the international conference series LSR (*Love and Sex with Robots*), initiated by David Levy (LSR1 2014 in Funchal, Portugal; LSR2 2016 in London, United Kingdom; LSR3 2017 in London, United Kingdom; and LSR4 2019 in Brussels, Belgium). The Google Scholar citation count reveals a range from 0 to more than 500 citations, the latter for David Levy’s [1] seminal book *Love and Sex with Robots*. Heavily cited sex robot publications are often not peer reviewed. Regarding the timeline, the oldest sex robot publication identified in the databases and included in our review is a 1997 comment of a sociologist on the impact of future sex robots [71] that raises questions still discussed today. However, it is an outlier, with approximately 85% (83/98) of the sex robot publications having been published in the last 5 years (2015-2019; Figure 3).

**Table 2:** Amount and type of research on sex robots (N=98 included academic publications, based on literature search in August 2019).

Reference		Citation count^a^	Peer review	Academic discipline
**Sex robot conceptualization and theory (n=40)**				
	Adshade (2017) [72]	0		Economics
	Barber (2017) [73]	2		Creative arts, film, and media
	Bołtuć (2017) [74]	3		Philosophy
	Carpenter (2017) [75]	2		Human-technology interaction
	Cheok et al (2017) [26]	3		Pervasive computing
	Cox-George and Bewley (2018) [17]	6		Medicine
	Cranny-Francis (2016) [76]	1	✓	Gender studies
	Danaher (2017) [77]	4		Ethics and law
	Danaher (2017) [78]	3		Ethics and law
	Danaher et al (2017) [79]	8		Ethics and law
	Devlin (2015) [80]	13		Computer science
	Devlin (2018) [69]	7		Computer science
	Döring and Pöschl (2018) [11]	6	✓	Psychology
	Eggleton (2019) [81]	1		Medicine
	Evans (2010) [82]	10		Robotics
	Facchin et al (2017) [83]	6		Clinical psychology
	Goldfeder and Razin (2015) [84]	7	✓	Law and religion
	Gutiu (2016) [85]	7		Law
	Hall (2017) [86]	2		Computer science
	Hauskeller (2017) [87]	1		Philosophy
	Herzfeld (2017) [88]	1		Science and religion
	Klein and Lin (2018) [89]	1		Technology ethics
	Kolivand et al (2018) [90]	1		Computer science
	Lee (2017) [70]	11		Media studies
	Levy (2007) [1]	531		Artificial intelligence
	Levy (2017) [91]	5		Artificial intelligence
	Mackenzie (2018) [24]	4	✓	Law and medical ethics
	McArthur and Twist (2017) [68]	11	✓	Philosophy
	Migotti and Wyatt (2017) [92]	0		Philosophy
	Musiał (2019) [93]	0		Philosophy
	Nyholm and Frank (2017) [94]	8		Philosophy
	Pearson (2015) [2]	8		Futurology
	Richardson (2016) [13]	77	✓	Social anthropology
	Rousi (2018) [95]	1		Cognitive science
	Rousi (2018) [96]	0	✓	Cognitive science
	Sharkey et al (2017) [97]	35		Computer science
	Snell (1997) [71]	9		Sociology
	Søraa (2017) [98]	9	✓	Interdisciplinary studies of culture
	Wennerscheid (2018) [99]	0		Literary studies
	Yeoman and Mars (2012) [15]	89	✓	Tourism management
**Ethics of sex robots (n=28)**				
	Amuda and Tijani (2012) [100]	14	✓	Law and theology
	Bendel (2015) [101]	23		Technical philosophy
	Bendel (2017) [102]	9		Technical philosophy
	Beschorner and Krause (2018) [103]	1		Business ethics
	Carvalho Nascimento et al (2018) [104]	0	✓	Bioethics
	Coeckelbergh (2009) [105]	62	✓	Philosophy of media and technology
	Di Nucci (2016) [106]	3		Ethics
	Di Nucci (2017) [107]	3		Ethics
	Frank and Nyholm (2017) [108]	13	✓	Philosophy and ethics
	Goldstein (2017) [109]	0		Political science
	González-González et al (2019) [110]	0		Gender studies
	Levy (2012) [14]	27		Artificial intelligence
	Mackenzie (2014) [111]	6		Law and medical ethics
	Mackenzie (2018) [23]	1	✓	Law and medical ethics
	McArthur (2017) [112]	2		Philosophy
	Petersen (2017) [113]	2		Philosophy
	Richardson (2016) [114]	2		Social anthropology
	Richardson (2016) [115]	27	✓	Social anthropology
	Russell (2009) [116]	8	✓	Law
	Shen (2019) [117]	2		Law
	Simmons (2016) [118]	1	✓	Law
	Sparrow (2017) [119]	25	✓	Philosophy
	Spencer (2011) [120]	1		Theology
	Sullins (2012) [121]	75	✓	Philosophy
	Wagner (2018) [122]	0		Robotics
	Welsh (2015) [123]	—^b^		Robot ethics
	Whitby (2012) [124]	0		Philosophy and ethics
	Ziaja (2011) [125]	7		Law
**Empirical studies on sex robot use and effects (n=12)**				
	Appel et al (2019) [126]	0	✓	Psychology
	Bartneck and McMullen (2018) [67]	3		Human-computer interaction
	Edirisinghe and Cheok (2017) [127]	2		Human-robot interaction
	Edirisinghe et al (2018) [128]	1		Human-robot interaction
	Korn et al (2018) [129]	0		Human-computer interaction
	Richards et al (2017) [130]	8		Communication
	Scheutz and Arnold (2016) [131]	55		Computer science
	Scheutz and Arnold (2017) [132]	2		Computer science
	Szczuka and Krämer (2017) [133]	2		Psychology
	Szczuka and Krämer (2018) [134]	1	✓	Psychology
	Szczuka and Krämer (2019) [135]	0	✓	Psychology
	Yulianto and Shidarta (2015) [136]	4	✓	Human-robot interaction
**Sex robot representations in art and media (n=8)**				
	Barber (2009) [137]	6		Creative arts, film, and media
	Beggan (2017) [138]	0	✓	Sociology
	Conn (2017) [139]	0		Comparative literature
	Döring and Poeschl (2019) [27]	0	✓	Psychology
	Gevers (2018) [140]	0		Art
	Hasse (2019) [141]	1	✓	Anthropology
	Hauskeller (2014) [142]	29		Philosophy
	Hawkes and Lacey (2019) [143]	0	✓	Media studies
**Legal regulation of child sex robots (n=6)**				
	Behrendt (2018) [18]	3		Philosophy
	Chatterjee (2019) [52]	0	✓	Criminology
	Danaher (2017) [144]	33	✓	Ethics and law
	Danaher (2019) [145]	0	✓	Ethics and law
	Maras and Shapiro (2017) [20]	7		Criminology and law
	Strikwerda (2017) [146]	5		Law and ethics
**Design of sex robots (n=4)**				
	Bendel (2018) [147]	3		Technical philosophy
	Danaher (2017) [148]	1		Ethics and law
	Gomes and Wu (2018) [9]	0	✓	Engineering
	Su et al (2019) [48]	0	✓	Human-computer interaction

^a^Citation count according to Google Scholar in August 2019.

^b^Google Scholar did not list the reference.

#### Research Findings on Sex Robots

In the following sections, the main findings of previous sex robot research will be reported separately for the 6 groups of sex robot publications (Table 2).

#### Sex Robot Conceptualization and Theory

The largest group of sex robot publications (40/98, 41%) deals with the conceptualization of sex robots and of human–sex robot relationships. Within this group, 2 issues are predominant: the (non)inherent sexism of sex robots and the (non)humanness of sex robots.

Echoing the critical feminist conceptualization of female sex dolls, several publications on sex robots characterize the female sex robot as an *inherently sexist object*. The most cited author of this position is Kathleen Richardson [13], who conceptualizes the female sex robot as a representative or surrogate of a sexually objectified woman, a female (forced) porn actor, a female (forced) prostitute, or a female sex slave. Following this conceptualization, the production and use of female sex robots is regarded as harmful for individual male users, their female partners, and society at large, as female sex robots symbolically reinvent and reaffirm the status of women as sex slaves [85]. However, this conceptualization operates more with metaphorical equations than established theories and is challenged by other publications as vague and unconvincing [79,80,89]. Although existing sex robots might appear sexist, different designs are possible; therefore, sex robots are not inherently sexist, according to other authors [11,69,76,78,98].

Further theoretical publications deal with the question of the humanness of the sex robot. Several publications stress that, by their definition, sexual interactions and intimate relationships are bidirectional and require a consenting human partner. Humanness—by the definition of these publications—implies sentience, first-person consciousness, and free will; none of these attributes can be ascribed to current sex robots. Consequently, the authors conclude that current sex robots are *nonhuman pseudo persons*. Accordingly, relations with robots are only *pseudorelationships* that inherently lack mutual concern for the welfare of each other [74] and do not lead to personal or spiritual growth [88]. Following this conceptualization, there is no sexual interaction possible *with* a robot or *between* a human and a robot, only robot-enhanced solipsistic masturbation [83].

There are also publications that focus on *future advanced sex robots* and their humanness. Several authors assume that, in the foreseeable future, sex robots could be produced that are sentient, self-conscious, and have a free will [24]. They might even have the legal status of citizens so that humans can legally marry them [72,84,91]. Such advanced humanoid robots will be so human-like that they must be conceptualized as persons and relations with them as interpersonal relationships. Advanced sex robots with excellent social and sexual skills and perfect looks who enter relationships with humans out of their own free will could be very attractive for many people [82]. Advanced sex and love robots could bring more love to the world [94], but they could, at the same time, devalue real humans [93]. However, the concept of an advanced sex robot that is almost indistinguishable from a real human paradoxically makes it seem pointless to build sex robots. If the advanced sex robot acts like a self-determined, willful human, if it consequently lies, cheats, criticizes, disregards, rejects, and leaves the human, what is the merit of creating it in the first place [87,92,95,96]?

Obviously, there is an inherent tension in the conceptualization of the degree and quality of humanness of interactive humanoid robots. A dumb robot is easy to control but lacks autonomous capabilities and sociability; hence, it cannot bring much additional value to traditional sex dolls. An intelligent advanced robot provides true sociability but lacks the manageability and obedience that we expect from a service technology.

A third conceptualization overcomes the divide between a sex robot as a mere masturbation aid and a sex robot as a quasihuman and stresses that successful sex robots can easily be imagined as purposefully designed to be nonhuman like regarding appearance, functionalities, and social role. Possibilities might be sex robots as synthetic animals [77], as fantasy creatures, or as interfaces to other types of sexual entertainment technology (*fantasy hardware*) [86]. Sex robots could be cherished and desired by humans, particularly by *digisexuals* or *technophilics*, precisely because of their *fundamental otherness* [68,99]. As humans can bond with sex dolls, it is even easier for them to form meaningful emotional attachments with interactive sex robots [26,73,75]. Instead of insisting that advanced sex robots be as human-like as possible to legitimize sexual interactions and emotional attachments with them, robots could also be accepted as nonhuman social agents, for example, to provide safe sex work [15], alleviate social and sexual deprivation [81], or allow for safe explorations of sexual fantasies [2].

The different implications of a wider use of sex robots are addressed by many theoretical publications as unanswered questions [71,97], for example, regarding health [17], social norms [70], and religious beliefs [90].

#### Ethics of Sex Robots

What is the *right thing* to do in view of the emergence of sex robots? The second largest group of sex robot publications (28/98, 29%; Table 2) attempts to tackle this core question of sex robot ethics. Although some authors stick to metareflection and debate which ethical approach to use [104], other authors provide answers of 3 different types:

- *Sex robots should not be built and used at all*. Starting from the assumption that human-human sexual and romantic relationships are most healthy and ethically superior to all human-robot pseudorelations and to the use of sexualized and sexist robotic objects, authors with different political [109], philosophical [119], feminist [114,115], theological [100,120], engineering [122], and legal [118] academic backgrounds reject further developments in this field. They call for bans and boycotts, stigmatization of, and abstinence from sex robots. Quite popular and often quoted in the media are the arguments of the earlier mentioned Kathleen Richardson, founder of the *Campaign against sex robots*, who compares sex robots with killer robots and with female sex slaves [114,115].
- *Sex robots should be built in an ethical way to avoid harm to humans, especially vulnerable humans*. Starting from the assumption that sex robots can be a good thing if they alleviate loneliness and/or sexual deprivation and contribute to the sexual and social well-being of individuals and couples, authors with different backgrounds encourage ethical design [105]. Publications are very diverse and often vague as to what exactly they expect from ethical sex robot design. One author explains that she wants sex robots designed in such a way that they do not get involved in acts of infidelity because they have learned the concept of heartbreak [125]. Other authors explicitly do not want sex robots to be *love robots* because they fear humans could be too easily manipulated by robots that fake romantic attachment [121,123]. Others want sex robots designed in a women-friendly [110] and disability-inclusive [106,107] way or demand design that is more focused on consumer safety [117]. Some authors point out the many different questions for ethical design ranging from “Should the robot become active on its own and entice the partner to have sex?” to “How should the robot collect an evaluate patient data to better satisfy its partner’s sexual needs?” [101,102]. As some authors assume that the development of sex robots is driven by a profit-oriented *uncaring industry* [124], there is a need for more involvement of ethically responsible entrepreneurs and designers from different backgrounds who aim to develop and market sex robots for sexual well-being, pleasure, fun, and play while taking into consideration the concerns and desires of diverse user and stakeholder groups. Some authors are very optimistic that sex robots will bring a lot of pleasure and happiness and are, therefore, ethically a good thing [112], although some ethical issues are unresolved (eg, regarding *robot prostitutes*) [14]. Other authors stress that robots are a good thing only for a very small group of people who absolutely cannot find a human sex partner [124].
- *Sex robots should be built in an ethical way to avoid harm to robots, especially for advanced sentient robots*. Starting from the assumption that future humanoid robots will be advanced to a very high degree of human likeness, according to several authors, their sexual and other citizen’s rights must be protected with a *nonanthropocentric* but *robocentric* ethic [23,24,103,111,116]. For example, sex with an advanced sex robot should only be acceptable if the robot has given explicit consent [108]. Although some authors stress the relevance of a robocentric ethic for sex robots to protect them from anticipated harm and exploitation, other authors argue that sentient robots designed as sex robots could have a “good life” and experience pleasure and satisfaction from fulfilling their tasks [113].

#### Empirical Studies on Sex Robot Use and Effects

The third group of sex robot publications contains empirical studies (12/98, 12%; Table 2). Thus far, not a single empirical study has been published that deals with the small but presumably growing number of pioneer users of sex robots. All existing studies address nonusers and investigate their attitudes toward sex robots and their reactions to sex robot–related stimuli.

Most empirical studies (8/12, 67%) are small web-based surveys on sex robot acceptance using convenience samples from the United States (N=261: [126], N=133: [130], N=100: [131], and N=198: [132]), Germany (N=263: [133]), Indonesia (N=380: [136]), and Malaysia (N=32: [127]). Their results show diverse rates of sex robot acceptance. For example, 40% of male and 17% of female respondents in the United States (mean age, 33 years) reported willingness to try out a sex robot [131] in comparison with 16% of Indonesian respondents [136] and 9% of Malaysian respondents [127]. Cultural background, male gender, positive attitudes toward robots in general, interest in manga and games, sensation seeking, and shyness appeared to be predictors of sex robot acceptance. Interestingly, sexual and relationship satisfaction did not predict sex robot acceptance [130,133]. However, because of the small nonrepresentative samples, the generalizability of existing sex robot acceptance data is very limited. Another problem is the varying operational definitions of sex robots given to respondents in the surveys. A Delphi survey explored the predictions of 20 social robot experts who were reluctant regarding sexual apps [129], whereas 1 expert interview explored the sex robot predictions of the founder of sex doll and sex robot manufacturer Abyss Creations, Matthew McMullen [67].

In addition to the survey and interview studies, 3 experimental studies were found (3/12, 25%). They investigated how heterosexual women experience their male partner’s imagined infidelity with a female robot vs a real woman [134], at which body parts of female robots vs female humans, both represented in pictures, male and female gaze [135], and how humans physically react when they touch different, including private, body parts of a robot that is not a sex robot [128]. Overall, these experiments show differences and similarities in humans’ sexuality-related reactions to humanoid robots and fellow humans. So far, no experimental study exists that uses an actual sex robot as the stimulus material.

#### Sex Robot Representations in Art and Media

The fourth group of sex robot publications concerns sex robot representations in art and media (8/98, 8%; Table 2). In their selected and analyzed examples from the science fiction literature, some studies from humanities focus on fictional female sexbots that seem to embody male fantasies of the ideal woman but who, in the course of the action, become *feminist robots* striving for independence from their male human partner or creator by leaving or even killing him, for example, the robot *Ava* in the 2015 UK movie *Ex Machina* [141,143], the virtual *Samantha* in the 2013 US movie *Her* [143], or the robotic wife in the 1981 Chinese story *Conjugal Happiness in the Arms of Morpheus* [139]. The famous US television series *Star Trek Voyager* presented the character *Seven of Nine*, a cybernetic organism and former Borg drone, who—although embodying traditional feminine beauty—challenged traditional ideas of gender and sexuality [137]. Other dystopian science fiction representations, selected and analyzed by the academic literature, illustrate the female sex robots’ sexual exploitation and victimization, for example, as porn actors in the 2009 US movie *2040* [138] or as rape victims in the US television series *Westworld* [143]. One monograph critically analyzes posthuman utopias in sex robot representations [142], and one editorial volume documents the *Robot Love 2018* International Expo of the *Niet Normaal Foundation* in the Netherlands that brought together researchers and artists [140].

A quantitative media content analysis examined the representation of human–sex robot relationships in 370 fictional and 340 nonfictional media examples [27]. The results of this study indicate that media representations of intimate human-robot relationships tend to portray the human partner as a man who is disadvantaged in interpersonal relationships. At the same time, media often portray the involved robot partner as a humanoid female sex robot. Although nonfictional media describe intimate human-robot relationships more often in sexual terms, fictional media focus more on emotional aspects, cohabitation, and even procreation between humans and robots. Overall, media representations of intimate human-robot relationships reveal stereotypical gender roles, heteronormativity, and a focus on sexual vs emotional intimacy [27].

#### Legal Regulation of Child Sex Robots

The fifth group of sex robot publications covers child sex robots (6/98, 6%; Table 2). All the 6 publications [18,20,52,144-146] characterize child sex robots as harmful and unethical and call for a legal ban that is already in preparation or in effect in several countries (eg, the aforementioned *CREEPER Act of 2017* in the US) [20]. In all, 2 publications speculate on the possible therapeutic uses of child sex robots. Although one of them assumes that their exploration would be too risky [145], the other encourages their exploration only in certain, controlled circumstances under strict medical supervision and in accordance with guidelines issued by an ethics committee [18].

#### Design of Sex Robots

Only 4 publications in the sex robot literature focus mainly on design (4/98, 4%; Table 2): 1 on erotic voice output [147], 1 on a mind-controlled neurodildo to be used separately or implemented in robots [9], 1 on general design aspects based on results about sex doll use [48], and 1 on feminist sex robot design in an analogy of initiatives for feminist pornography [148].

#### Research Gaps in Sex Robots

Although the body of sex robot publications is 3 times as large as that of sex doll publications, *empirical* studies on sex robot use and effects are equally scarce (4 peer-reviewed papers in total). Fundamental questions regarding the sexual use of human-like full-body material artifacts that remained unanswered for sex dolls also remain unanswered for sex robots.

#### Research Gaps in Sex Robot Design

Just as with sex dolls, the question of how much fantasy, and which and whose fantasies should legitimately be implemented in sexual fantasy products to make them socially acceptable, harmless, and still sexually desirable and exciting, remains unanswered with sex robots as well. Although there is a lot of speculation on the possible therapeutic uses of sex robots to be found in public and academic debates, the literature fails to provide design guidelines for therapeutic sex robots informed by evidence from sex and relationship therapy and focused on specific problems (eg, on sexual shyness and anxiety, sexual dysfunctions, sexual trauma, paraphilias, and paraphilic disorders). Design studies for current sex robots hardly exist, and the literature predominantly speculates about imagined future sex robots. Instead of researchers, it was journalists who first dealt with the question, “What would sex robots for women look like?” [149] and who let women and men draw and explain their *ideal sex robots* [150].

#### Research Gaps in Sex Robot Use

Thus far, no empirical study has investigated experienced sex robot users or interactions of unexperienced participants with actual sex robots. Results from research on sex doll use are, therefore, the best available proxy for sex robot use. Regarding the potential market size and user population, there are also no data available that allow for sound predictions. One might speculate that sex robots could overcome some of the stigmatization of sex dolls as sex robots can be framed as cutting edge, high-tech products. Thus, their users might appear more modern, future oriented, and competent in comparison with traditional sex doll owners. Against this backdrop, one would expect more growth for the sex robot market than for the sex doll market, but data are needed. To further explore the sexual appeal of robots, insights from research on objectophilia [151,152] and technofetishism [153] could be helpful.

Surprisingly, the sex robot literature falls short in conceptualizing and investigating interactions and relationships between humans and current sex robots in a psychologically nuanced way. Whereas the sex doll literature has already established that dolls easily trigger humans to build meaningful, caring, loving, long-term relationships with them, the sex robot literature often falls back on binary thinking. It categorizes the current sex robot as an inanimate object and mere masturbation aid without any sociability and is only willing to ascribe sociability to future imagined sex robots that are advanced to the point of indistinguishability from humans. Hence, the literature on sex robots often misses the key point that robots are more than mere masturbation aids due to anthropomorphization and that they are meaningful and possibly helpful precisely because they are not substitutes for real humans but are sociotechnical entities for parasocial use and play. Parasocial interactions and play with sexual fantasy products grant more degrees of freedom in sexual expression and allow to take a break from all of the norms, ethics, expectations, and responsibilities of human-human interactions.

#### Research Gaps in Sex Robot Effects

As sex robot users and use are completely unknown thus far, any claims about positive and/or negative effects are mere speculation. Although some authors are so convinced of their speculations on strong to catastrophic negative effects that they demand immediate boycotts and bans of sex robots, others urgently call for empirical research on sex robot effects. The idea that sex robots allow humans to indulge in interactive embodied sexual fantasies elicits strong projections of lust and fear. Most likely, empirical research will help us overcome exaggerated projections and understand the diversity and ambivalence of effects on different types of sex robot users.

## Discussion

### Main Results of the Review

In conclusion, the main results of the whole review are summarized, its limitations are indicated, and a roadmap for future research is drawn. The body of sex robot literature, with approximately 100 academic publications in total, is more than 3 times larger than that on sex doll literature, with approximately 30 publications (RQ1). However, only a handful of peer-reviewed empirical papers on both sex doll use and sex robot use are available thus far. No sex robot study exists that investigates people experienced in sex robot use and/or introduces actual sex dolls or sex robots as stimulus material. Regarding the first RQ, one must concede that sex dolls and sex robots, although attracting growing public and scholarly attention, are heavily under-researched. Both sex doll and sex robot research are fields characterized by disciplinary diversity, with notable participation from philosophy, humanities, and engineering, and a conspicuous lack of participation from sex researchers.

Sex doll and sex robot designs (RQ2) are often critically assessed in the literature, mainly because the bodies of women-like dolls and robots are usually designed in sexualized ways following and exaggerating traditional feminine beauty ideals. However, when understanding sex dolls and sex robots as sexual fantasy products, it makes sense that they do not imitate reality but cater to sexual fantasy. Often, it is exactly the point of sexual fantasies to be unrealistic. Thus far, the literature has not addressed the core question of how we could and should assess designs of sexual fantasy products such as sex dolls and sex robots, considering both social inequalities and vulnerabilities and the freedom of sexual fantasy and expression. Regarding future advanced sex robots, the literature presents various requirements for ethical design, which—at the current state of robot development—are very speculative. Systematic design studies that work with current and future users (eg, private sex doll owners, sex workers, and sex therapists) and address different use scenarios (eg, domestic, commercial, or therapeutic) are lacking.

Although previous research has provided some insights into the domestic long-term use of sex dolls (with or without parallel psychotherapy), no data have been collected thus far on the short-term interactions or long-term relationships between humans and sex robots. Thus, the best proxy for sex robot use and users today is the limited data on sex doll use and users (RQ3).

Considering the lack of empirical knowledge about sex doll users and sex robot users and use, it is obvious that the predictions of positive and negative effects found in the literature can only be speculative (RQ4). It is striking that authors still provide very strong and contradictory effect claims ranging from utopian visions of improved sexual satisfaction and overall happiness to dystopian visions of dehumanization, objectification, and isolation. Predictions of small and/or ambivalent effects might be more realistic but are seldom discussed in the academic literature thus far, which seems to mirror some of the hype and scandalization observable in public media discourses.

### Limitations

This scoping review addressed sex doll and sex robot research as far as it is represented in the accessible literature published before August 2019. We were particularly careful to retrieve publications not only from the databases but also, in a systematic way, from all the included publications’ reference lists. Nevertheless, it must be taken into consideration that further studies that have not (yet) been published and/or were not (yet) accessible (eg, conference presentations, qualifications theses, and journal articles under peer review) could exist. However, we are confident that our systematic literature identification strategy covered previous research thoroughly enough, especially as this is the very first systematic review of the field.

To map previous research in a comprehensible and useful manner, we organized the body of literature by building distinct groups of publications according to their key topics and methodologies. We discussed data charting and synthesis within the team and checked everything in duplicate. However, some decisions might be questionable. There is an inherent tension between the aim of providing a clear and comprehensible structure, which requires a reduction in complexity, and the aim of doing justice to the individual publications, which requires a representation of their complexities. Due to space constraints, we were forced to reduce the complexity much more than we would have wished. Therefore, we encourage readers to consult the original publications whenever in doubt and apologize to fellow researchers in case they feel our review misrepresents their work.

Another inherent problem of a multidisciplinary review lies in the tension between the aim of doing justice to discipline-specific styles of knowledge production and communication and the aim of presenting existing knowledge in a consistent, readable, and generally understandable way. We deliberately simplified concepts and streamlined discipline-specific jargon to improve consistency. We provide a broad overview spanning from ancient Greek myths to contemporary web-based surveys, and spanning from the psychoanalyst’s office to the robotics lab. We agree with many authors we cite in this review that a deeper understanding of sex dolls and sex robots and their meanings for human sexuality can only be achieved through more interdisciplinary collaboration. We hope that our prioritization of disciplinary width over depth will inspire this collaboration. However, we are aware of the risks and limitations of simplification.

### Roadmap for Future Research

Hopefully, the many and diverse research gaps pointed out in this review can serve as starting points for future research projects on sex dolls and sex robots, their design, use, and effects. To conclude, we suggest 4 selected, particularly urgent research strands:

1. *Public debates* about and *media representations* of sex dolls and sex robots, polarized and scandalizing as they are, attract much attention, shape public opinions, and influence research activity. They deserve more scholarly analysis and participation by the sex research community. This includes traditional mass media and social media. Mass media tend to assume dramatic positive or negative effects, while often completely ignoring the fact that sex dolls and sex robots, overall, could have only small and/or ambivalent effects. Social media, sometimes, offer more nuanced views, but expert statements and documentaries about sex robots on YouTube, for instance, are met by a noteworthy amount of misogynist comments that welcome female sex robots as substitutes for women. On Twitter and Instagram, we see sex dolls communicating to the public, their accounts steered by doll owners (eg, the earlier mentioned *Davecat*) and by doll manufacturers. These examples illustrate that we need to know more about media representations as they are an important element of the cultural context in which sex dolls and sex robots are developed, marketed, discussed, used, and investigated today.
2. Research on the sexual uses of human-like *material artifacts* such as sex dolls and sex robots needs to be advanced and connected to research on human-like *digital artifacts* such as chat bots, avatars, holograms, or immersive virtual reality pornography. After all, a sexual AI system trained by a particular user could be used on different technological and media platforms such as a full-body sex robot, an immersive virtual reality system, or a smartphone. Although the materiality of dolls and robots offers new possibilities in terms of embodied sexual fantasies (eg, physical presence, physical care, physical touch, and physical stimulation), it also creates boundaries (eg, through the body weight and difficult handling of the dolls and robots at home, limited mobility outdoors, high visibility, and risk of social stigmatization). For sexual fantasy products that aim to enhance their users’ sexual and social experiences, the right degree and mixture of materiality and virtuality is an open question for research and design.
3. Although sex robots have triggered the publication of many theoretical and ethical papers, we urgently need *empirical data* on actual sex doll and sex robot users and uses. Different study designs (nonexperimental and experimental, cross-sectional, and longitudinal) and data collection methods (qualitative interviews, focus group discussions, surveys, psychological tests, and physiological measurements) are suitable for research with actual sex doll and sex robot users. Instead of using only imagined or visually depicted artifacts as stimulus material, some of the real sex dolls and sex robots should be incorporated in empirical studies.
4. Despite the relatively large number of theoretical papers, the degree of *theoretical elaboration* of human–sex doll/sex robot relations and their consequences is not yet very high [12]. Commonly used theoretical concepts are objectification, gratification, and pseudorelationships. For a more thorough understanding, we suggest including theoretical concepts from the field of doll play and doll therapy and from the field of human interaction with media personas (parasocial interactions and relationships), with digital technologies (computers as social actors and media equation theory) and with social robots (uncanny valley concept and anthropomorphization) as well as from social psychology (social cognitive learning theory), clinical and developmental psychology (transitional objects, objectophilia, and robophilia), and sexuality research (sexual scripts theory and theories on sexual fantasies). It is not yet clear which theories from the different related research fields on dolls, robots, sexuality, gender relations, well-being, and health are best applicable to human–sex doll/robot relationships and if and how they can be combined to best explain the complex intimate engagements of humans with artifacts.

## Appendix

Multimedia Appendix 1 Literature searches as of August 6 to 9, 2019.

## Abbreviations

AI: artificial intelligence
BBC: British Broadcasting Corporation
CREEPER: Curbing Realistic Exploitative Electronic Pedophilic Robot
PRISMA-ScR: Preferred Reporting Items for Systematic Reviews and Meta-Analyses extension for Scoping Reviews
RQ: review question
