# Supplementary material for: Design, Use, and Effects of Sex Dolls and Sex Robots: Scoping Review
Source: J Med Internet Res. 2020 Jul 30;22(7):e18551. doi: 10.2196/18551 (PMC7426804; doi:10.2196/18551)
Supplement: Multimedia Appendix 1 [file jmir_v22i7e18551_app1.doc]

## Multimedia Appendix 1: Literature searches as of 6^th –^ 9^th^ of August 2019

1. Scopus

1.1 (TITLE-ABS-KEY ("sex doll")  OR  TITLE-ABS-KEY ("sex dolls")  OR  TITLE-ABS-KEY ("doll sex")  OR TITLE-ABS-KEY ("love doll")  OR  TITLE-ABS-KEY ("love dolls")  OR  TITLE-ABS-KEY ("doll love")) AND  (LIMIT-TO (LANGUAGE,  "English" )) 
→ 24 hits (2011-2019)

1.2 (TITLE-ABS-KEY ("sex robot")  OR  TITLE-ABS-KEY ("sex robots") OR TITLE-ABS-KEY ("sexbot") OR  TITLE-ABS-KEY ("sex bot") OR TITLE-ABS-KEY ("robot sex") OR TITLE-ABS-KEY ("love robot") OR  TITLE-ABS-KEY ("love robots") OR  TITLE-ABS-KEY ("lovebot") OR  TITLE-ABS-KEY ("love bot") OR TITLE-ABS-KEY ("robot love"))  AND  (LIMIT-TO (LANGUAGE,  "English"))
→ 73 hits (1984/2006-2019)

2. MEDLINE

2.1 TI = ("sex doll" OR "sex dolls" OR "doll sex" OR "love doll" OR "love dolls" OR "doll love") [AND LANGUAGE: (English)]
→ 0 hits

2.2 TI = ("sex robot" OR "sex robots" OR "sexbot" OR "sex bot" OR "robot sex" OR "love robot" OR "love robots" OR "lovebot" OR "love bot" OR "robot love") [AND LANGUAGE: (English)]
→ 7 hits (2017-2019)

3. PsycINFO

3.1 (TI "sex doll" OR TI "sex dolls" OR TI "doll sex" OR TI "love doll" OR TI "love dolls" OR TI "doll love" OR AB "sex doll" OR AB "sex dolls" OR AB "doll sex" OR AB "love doll" OR AB "love dolls" OR AB "doll love") AND  LA English
→ 5 hits (2009-2018)

3.2 (TI "sex robot" OR TI "sex robots" OR TI "sexbot" OR TI "sex bot" OR TI "robot sex" OR TI "love robot" OR TI "love robots" OR TI "lovebot" OR TI "love bot" OR TI "robot love" OR AB "sex robot" OR AB "sex robots" OR AB "sexbot" OR AB "sex bot" OR AB "robot sex" OR AB "love robot" OR AB "love robots" OR AB "lovebot" OR AB "love bot" OR AB "robot love") AND  LA English
→ 7 hits (2005-2019)

4. IEEE Xplore

4.1 ("Document Title": "sex doll") OR ("Document Title": "sex dolls") OR ("Document Title": "doll sex") OR  ("Document Title": "love doll") OR ("Document Title": "love dolls") OR ("Document Title": "doll love")  OR ("Abstract": "sex doll") OR ("Abstract": "sex dolls") OR ("Abstract": "doll sex") OR ("Abstract": "love doll") OR ("Abstract": "love dolls") OR ("Abstract": "doll love") 
→ 2 hits (2012-2017)

4.2 ("Document Title": "sex robot") OR ("Document Title": "sex robots") OR ("Document Title": "sexbot") OR ("Document Title": "sex bot") OR ("Document Title": "robot sex") OR ("Document Title": "love robot") OR ("Document Title": "love robots") OR ("Document Title": "lovebot") OR ("Document Title": "love bot“) OR ("Document Title": "robot love“) OR ("Abstract": "sex robot") OR ("Abstract": "sex robots") OR ("Abstract": "sexbot") OR ("Abstract": "sex bot") OR ("Abstract": "robot sex") OR ("Abstract": "love robot") OR ("Abstract": "love robots") OR ("Abstract": "lovebot") OR ("Abstract": "love bot“) OR ("Abstract": "robot love bot“)
→ 23 hits (2012-2018)

5. ACM Digital Library – Guide to Computing Literature

5.1 acmdlTitle:(+"sex doll") OR acmdlTitle:(+"sex dolls") OR acmdlTitle:(+"doll sex") OR acmdlTitle:(+"love doll") OR acmdlTitle:(+"love dolls") OR acmdlTitle:(+"doll love") OR recordAbstract:(+"sex doll") OR recordAbstract:(+"sex dolls") OR recordAbstract:(+"doll sex") OR recordAbstract:(+"love doll") OR recordAbstract:(+"love dolls") OR recordAbstract:(+"doll love")
→ 3 hits (2011-2019)

5.2 acmdlTitle:(+"sex robot") OR acmdlTitle:(+"sex robots") OR acmdlTitle:(+"sexbot") OR acmdlTitle:(+"sex bot") OR acmdlTitle:(+"robot sex") OR acmdlTitle:(+"love robot") OR acmdlTitle:(+"love robots") OR acmdlTitle:(+"lovebot") OR acmdlTitle:(+"love bot") OR acmdlTitle:(+"robot love") OR recordAbstract:(+"sex robot") OR recordAbstract:(+"sex robots") OR recordAbstract:(+"sexbot") OR recordAbstract:(+"sex bot") OR recordAbstract:(+"robot sex") OR recordAbstract:(+"love robot") OR recordAbstract:(+"love robots") OR recordAbstract:(+"lovebot") OR recordAbstract:(+"love bot") OR recordAbstract:(+"robot love")
→ 27 hits (1993-2019)
